# Characterizing healthcare personnel attitudes toward receipt of a voluntary bivalent COVID-19 booster vaccine during a COVID-19 outbreak at a behavioral health hospital in Connecticut

**DOI:** 10.1017/ash.2024.78

**Published:** 2024-05-16

**Authors:** Scott C. Roberts, Kathryn Willebrand, Jacqueline Fredrick, Lauren Pischel, Kavin Patel, Thomas S. Murray, Richard A. Martinello

**Affiliations:** 1 Section of Infectious Diseases, Department of Internal Medicine, Yale School of Medicine, New Haven, CT, USA; 2 Infection Prevention, Yale New Haven Hospital, New Haven, CT, USA; 3 Section of Infectious Diseases, Department of Pediatrics, Yale School of Medicine, New Haven, CT, USA

## Abstract

COVID-19 vaccine uptake in healthcare personnel (HCP) is poor. A cross-sectional survey study of behavioral health HCP was performed. Commonly identified reasons for vaccination were protecting others and oneself. Reasons against were a lack of perceived protection, dosing intervals, and side effects. Assessing vaccination attitudes can assist in uptake strategy.

## Introduction

Vaccination is a key strategy to reduce the impact of COVID-19 on patients and healthcare personnel (HCP). Despite the effectiveness of the updated booster vaccines against COVID-19, a minority of eligible recipients have received a booster. In HCP working in acute care hospitals and nursing homes for the 2022–2023 season, just 17% and 23% were up to date with COVID-19 vaccination respectively, rates well below those of influenza vaccination.^1^ Prior studies have attempted to understand the reasons for poor uptake in the community, which have included a lack of awareness of eligibility and availability, as well as perceptions of sufficient immunity pre-vaccination.^2^ In this cross-sectional survey study, we investigated attitudes toward the bivalent booster uptake in a behavioral health hospital shortly after an outbreak of COVID-19 where the booster dose was offered to all HCPs.

## Methods

An observational cross-sectional survey study was performed at Yale New Haven Psychiatric Hospital in December 2022. A self-administered survey tool adapted from prior studies evaluating HCP perspectives on influenza and COVID-19 vaccination was developed in Qualtrics and sent via electronic communication on two occasions several weeks apart to all HCP.^3–5^ The survey queried self-identified demographic data including age category, sex, race, ethnicity, job category, history of COVID-19, prior COVID-19 vaccinations, perception of COVID-19 exposure risk, and updated/bivalent booster doses. Reasons for or against receipt of the bivalent booster dose were elicited, with the option of HCP selecting all reasons that applied to them, as well as providing optional free text response. The survey was administered several weeks after a COVID-19 outbreak in several of the behavioral health units that was ultimately controlled with multiple interventions including frequent testing of all inpatients and new admissions, and mandatory use of personal protective equipment for HCP. Receipt of the COVID-19 primary vaccination series and one booster dose were mandated for HCP, however, most HCPs fulfilled this requirement with the monovalent booster and only newly hired HCPs were mandated to receive the bivalent booster once it became available, otherwise receive was voluntary.

Statistical analysis was performed with IBM SPSS version 28.0. Categorical and continuous variables were analyzed with Fisher’s exact test and a two-tailed t-test respectively, with a p-value of <0.05 considered significant. This qualitative improvement initiative was exempt from Yale University Institutional Board Review.

## Results

The survey was sent to 664 HCPs with primary assignments in behavioral health settings and 182 (27.4%) provided complete responses to the survey. One hundred HCPs (55.0%) received the bivalent booster. Ninety-one HCPs (50.0%) reported previously having COVID-19 at least once. HCPs self-identifying as white were more likely to have received the bivalent booster dose (62.7%, *n* = 74 out of 118) compared with Black HCP (42.9%, *n* = 12 out of 28, *p* = 0.037). Otherwise, there was no difference in age, sex, or ethnicity. Although HCP working in healthcare for more years had higher proportions of booster receipt, when adjusting for age this was not significant (*p* = 0.168). There was no significant difference in who received the booster and who did not according to primary work setting (inpatient versus outpatient), perceived degree of workplace exposure to COVID-19, or patient-facing- versus non-patient-facing HCP. Additional data, including receipt by job classification, is found in the Table 1.

**Table 1.** Demographic data and characteristics of HCP who received and did not receive the bivalent COVID-19 booster vaccine

Variable	Received vaccine (*n* = 100)	Did not receive vaccine (*n* = 82)	*p*-value
**Age**			*p* = 0.062
18–24	0 (0.0%)	2 (2.6%)	
25–34	16 (16.3%)	11 (14.5%)	
35–44	20 (20.4%)	19 (25.0%)	
45–54	23 (23.5%)	26 (34.2%)	
55–64	30 (30.6%)	17 (22.4%)	
65–74	9 (9.2%)	1 (1.3%)	
**Sex**			*p* = 0.057
Female	76 (77.6%)	49 (64.5%)	
Male	22 (22.4%)9/	27 (35.5%)	
**Race**			*p* = 0.037
White	74 (78.7%)	44 (64.7%)	
Black or AA	12 (12.8%)	16 (23.5%)	
Asian	7 (7.4%)	3 (4.4%)	
Other	1 (1.1%)	5 (7.4%)	
**Ethnicity**			*p* = 0.455
Hispanic/Latino	10 (10.1%)	5 (6.8%)	
Not Hispanic/Latino	89 (89.9%)	68 (93.2%)	
**History of COVID-19**			*p* = 0.766
Yes	49 (49.0%)	42 (51.2%)	
No	51 (51.0%)	40 (48.8%)	
**Years in healthcare**			*p* = 0.053
0–1	1 (1.0%)	1 (1.2%)	
1–2	1 (1.0%)	4 (4.9%)	
2–5	7 (7.0%)	13 (15.9%)	
5–10	21 (21.0%)	12 (14.6%)	
10–20	25 (25.0%)	28 (34.1%)	
20+	45 (45.0%)	24 (29.3%)	
**Workplace exposure to COVID-19**			*p* = 0.814
Every shift	21 (21.0%)	17 (20.7%)	
Most shifts	11 (11.0%)	13 (15.9%)	
Some shifts	45 (45.0%)	31 (37.8%)	
Rarely	19 (19.0%)	18 (22.0%)	
Never	4 (4.0%)	3 (3.7%)	
**Work setting**			*p* = 0.222
Primarily inpatient	70 (70.0%)	58 (70.7%)	
Primarily outpatient	24 (24.0%)	14 (17.1%)	
Both equally	6 (6.0%)	10 (12.2%)	
**Patient facing**			*p* = 0.181
Yes	86 (86.0%)	75 (91.5%)	
No	14 (14.0%)	7 (85.4%)	
**Job classification**			*p* = 0.047
Physician (MD/DO)	12 (12.0%)	6 (7.3%)	
Nurse (RN, LPN)	16 (16.0%)	27 (32.9%)	
SW	24 (24.0%)	15 (18.3%)	
APP	13 (13.0%)	7 (8.5%)	
PCA	7 (7.0%)	11 (13.4%)	
Pharmacist	3 (3.0%)	1 (1.2%)	
Business associate	2 (2.0%)	1 (1.2%)	
Protective services	4 (4.0%)	6 (7.3%)	
Other	19 (19.0%)	8 (9.8%)	

*SW = social worker/therapist, PCA = patient care assistant.

**Figure 1.**
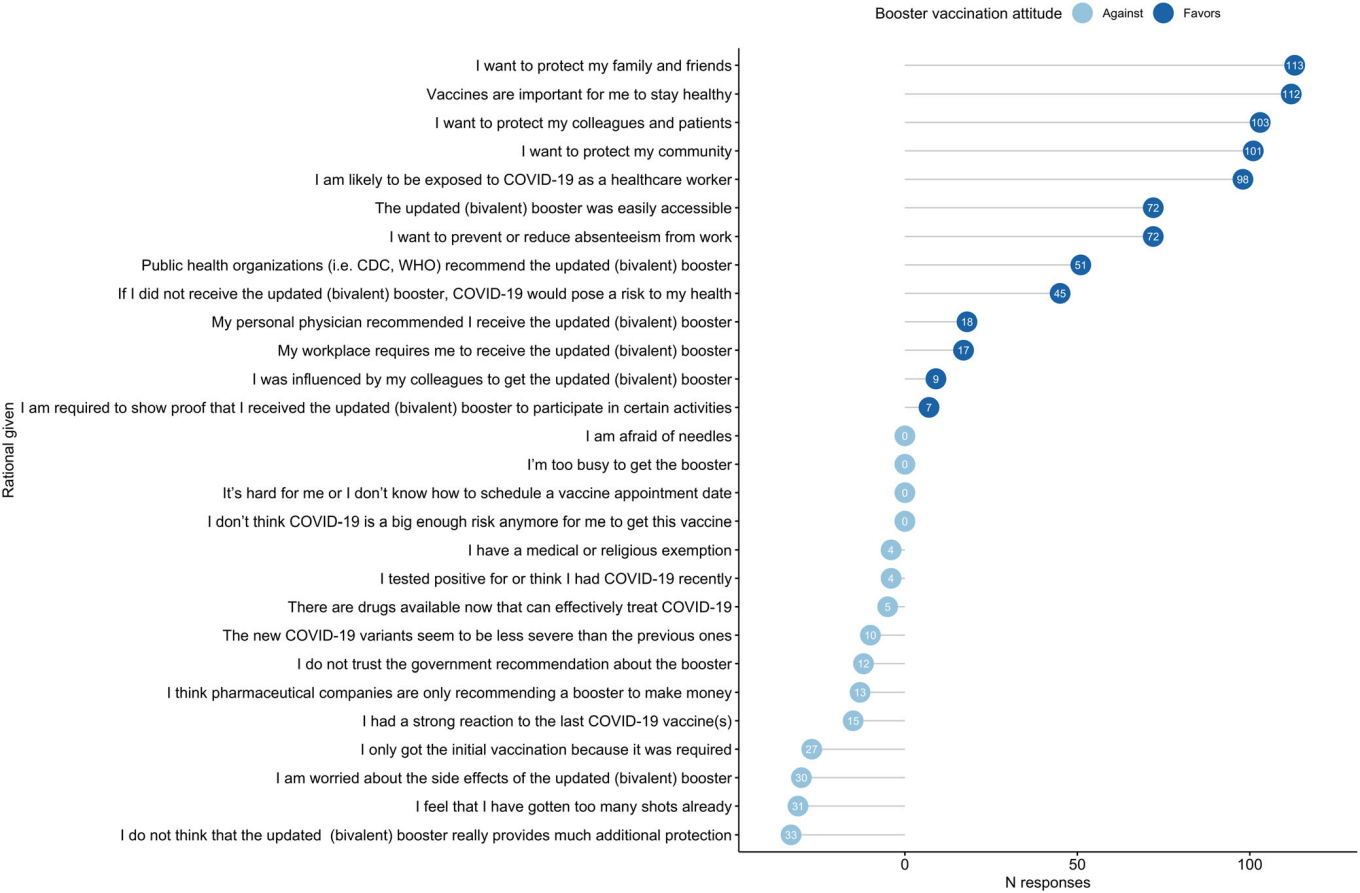
Reasons for or against receiving the bivalent booster dose.

The most identified reasons for receiving the bivalent booster among those who received or were planning to receive it included a “desire to protect family and friends” (*n* = 113), importance of “stay healthy” (*n* = 112), and “protecting colleagues and patients” (*n* = 103). The most identified reasons for not wanting to receive the bivalent booster dose were “not thinking it provides additional protection” (*n* = 33), “too many” shots already received (*n* = 31), and “concern about side effects” (*n* = 30) (Figure 1).

## Discussion

Bivalent booster dose uptake among HCPs working on inpatient behavioral health units shortly after a COVID-19 outbreak when offered voluntarily was greater than rates reported in HCPs nationally. HCP reported varying reasons for and against receipt of the bivalent booster dose, with the most common being protection of family and friends, and perceptions of no additional protection respectively. Prior studies have found the low uptake in the community to be due to a lack of awareness towards availability and eligibility, both of which were accounted for in this study as this was directly offered to the HCP.^2^ We observed altruism to be a major theme for vaccine receipt, with two of the three reasons most identified being protecting others at home and the workplace. A prior study found altruism-tailored communication had a positive impact on attitudes towards influenza vaccination, findings that should be explored with COVID-19 vaccination, especially with HCP.^6^

We found white HCPs were more likely to have received the bivalent booster when compared to Black HCPs, attitudes which have been observed in prior reports.^7^ Older HCPs, who are at higher risk for severe COVID-19, were more likely to receive the bivalent booster when compared to younger HCP; in our study, 90% of HCP over the age of 65 received the booster. We also observed differences in the rates of bivalent booster uptake according to job classification, with physicians, advanced practice practitioners, and social workers & behavioral health therapists having higher rates of uptake while other groups, including nurses and patient care associates, had lower rates, findings previously described in influenza vaccination.^8^ This is especially concerning, as nurses and the patient care assistants are considered the most “front line” of HCP due to increased patient contact, and thus have the highest risk of COVID-19 exposure.

Despite many HCPs reporting likely COVID-19 exposure in the workplace, there was no greater receipt of the bivalent booster in these individuals. We suspect this is reflective of the fact that many HCPs did not perceive additional protection from receiving the bivalent booster, which was the most stated reason for declination in our study. This may also have been compounded by the recency of working during a COVID-19 outbreak, as this study was administered only weeks after the outbreak was controlled, although only a select few HCPs reported recent COVID-19 infection as a reason for declination. COVID-19 outbreaks can have large impacts on patient care, admissions, and discharges especially in psychiatric wards, as well as limit psychiatric services and patient care offered in these units which are often communal in nature. Involvement in such disruptions could account for health care providers being more amenable to booster uptake than the general population. Assessing HCP attitudes toward the bivalent booster dose can assist in guiding communication and outreach strategies to increase vaccine uptake by HCP.

A limitation includes voluntary response bias where results are biased towards individuals more likely to receive a bivalent booster vaccine. It is unclear if reasons for declining the vaccine are representative of HCP who did not complete the survey, and we do not have data on HCPs who did not complete the survey. This was also performed shortly after a COVID-19 outbreak, which may have led to recency bias especially in those recently exposed or infected. We did not perform multivariable logistic regression, as the limited sample size precluded effective adjustment for confounding.
